# Performance Improvement of Time-Differenced Carrier Phase Measurement-Based Integrated GPS/INS Considering Noise Correlation

**DOI:** 10.3390/s19143084

**Published:** 2019-07-12

**Authors:** Jungbeom Kim, Younsil Kim, Junesol Song, Donguk Kim, Minhuck Park, Changdon Kee

**Affiliations:** 1 School of Mechanical and Aerospace Engineering and the Institute of Advanced Aerospace Technology, Seoul National University, Seoul 08826, Korea; 2 Korea Aerospace Research Institute (KARI), Daejeon 34133, Korea; 3 Ecole Nationale de l’Aviation Civile (ENAC), 31400 Toulouse, France

**Keywords:** GPS/INS, time-differenced carrier-phase, noise correlation

## Abstract

In this study, we combined a time-differenced carrier phase (TDCP)-based global positioning system (GPS) with an inertial navigation system (INS) to form an integrated system that appropriately considers noise correlation. The TDCP-based navigation system can determine positions precisely based on high-quality carrier phase measurements without difficulty resolving integer ambiguity. Because the TDCP system contains current and previous information that violate the format of the conventional Kalman filter, a delayed state filter that considers the correlation between process and measurement noise is utilized to improve the accuracy and reliability of the TDCP-based GPS/INS. The results of a dynamic simulation and an experiment conducted to verify the efficacy of the proposed system indicate that it can achieve performance improvements of up to 70% and 60%, respectively, compared to the conventional algorithm.

## 1. Introduction

The global positioning system (GPS) is widely employed to determine the position, velocity, and time (PVT) information of users on Earth based on signals broadcast through GPS satellites in space. However, GPS signals are ineffective in situations such as jamming and spoofing, and areas such as tunnels or where numerous buildings are in close proximity to each other. Low sampling rate, typically 1 Hz, is another drawback of GPS. The inertial navigation system (INS) has divergence errors that manifest after continuous use over an extended period, but it has a high sampling rate, typically 100 Hz, and is robust against external environmental factors. Considering the advantages of both systems, it is clear that combining GPS and INS should produce results that are superior to those that can be achieved using either system alone [1]. As the price of a low-cost GPS chipset has fallen below 1 USD and micro-electro mechanical system (MEMS) technology has reduced the INS cost considerably [2], there is significant interest in enhancing the accuracy of low-cost GPS/INS systems.

GPS/INS integration primarily involves using a Kalman filter (KF). There are three main types of integration, namely, loosely coupled (LC), tightly coupled (TC), and ultra-tight (UT) integration [3,4]. In LC integration, the positions and velocities estimated by the GPS receiver are combined with INS navigation solutions, while in the case of TC integration, raw GPS measurements such as pseudorange and Doppler observables are processed with the INS measurements to estimate the PVT information. In UT integration, an INS is used to help the GPS receiver capture and track GPS signals [4,5]. In this study, the simplest integration method, i.e., LC integration, was employed to investigate how time-differenced carrier phase (TDCP) measurements are utilized, rather than comparing the performances of these integration methods. 

Two kinds of measurements are employed to calculate position information using GPS receivers: pseudorange and carrier phase. Although pseudorange measurements have a higher noise level than carrier-phase measurements, they are predominantly used by low-cost GPS receivers because the positioning method is simple. In contrast, carrier phase measurements have low noise (on the order of millimeters) and thus can provide high accuracy. However, in order to use carrier-phase measurements, the integer ambiguity of the measurements has to be resolved. Several integer ambiguity resolution methods, such as least square ambiguity decorrelation adjustment (LAMBDA), currently exist, and some of these methods can be employed using only single-frequency GPS receivers; however, these methods require expensive dual-frequency GPS receivers for fast and accurate ambiguity resolution [6,7]. Furthermore, although the price of dual-frequency GPS receivers has been decreasing, they are still more expensive than single-frequency GPS receivers, which are used in the smartphones or vehicle navigation systems considered in this paper. Integer ambiguity is time-invariant and can therefore be eliminated through time differencing, with parameters such as hardware biases being removed [8,9]. Therefore, in this study, TDCP measurements were used to acquire more precise positioning information.

TDCP measurements have been widely employed for precise velocity estimation. In general, the velocities estimated by Doppler measurement have an accuracy of centimeters per second, whereas those based on TDCP have an accuracy of millimeters per second [9,10,11]. Thus, TDCP measurements can be used to estimate the amount of position change between two consecutive epochs more precisely than Doppler measurements. Therefore, extensive research is being conducted on methods to integrate TDCP measurements from low-cost GPS receivers and INSs to enhance positioning accuracy. TDCP measurements contain previous information, which violates the format of the usual KF model. In most studies, various approximations are used to accommodate the previous information and design new measurement equations in order to apply to the format of the usual KF model. Soon et al. [10] proposed an iterative extended KF to estimate position and clock drift using the linearity between consecutive epochs. Wendel et al. [12,13] also proposed a new TDCP/INS approach in which TDCP measurements are applied to the usual KF as velocity information. However, Han and Wang [14] asserted that the modeling and integration method for utilizing TDCP measurements described by Wendel were incorrect and proposed a different approach using a dual-rate KF with pseudorange and TDCP measurements. Zhao [15] reported that the TDCP measurement modeling methods employed in previous studies produced inaccurate results and proposed a different modeling method. While previous studies have dealt with TDCP measurements such as velocity information, Li et al. [5] estimated the relative position in terms of the phase-derived position increment (PDPI) using TDCP measurements and improved the positioning accuracy by applying additional PDPI measurements in the conventional TC method. However, these studies simply dealt with how to model TDCP measurements, as well as the accuracy of the results, without addressing reliability issues, such as covariance bounding. In addition, Travis [16] summarized the equations considering the correlated process and measurement noise of the TDCP/INS approach, but the equations related to the correlated noise were not derived correctly and thus do not permit the optimal solution to be obtained.

In this paper, we propose a TDCP-based integrated GPS/INS considering noise correlation. A delayed state filter that considers correlated process and measurement noise is utilized to improve the accuracy and reliability of the GPS/INS using TDCP measurements, unlike in previous methods. As discussed previously, TDCP measurements consist of information from two consecutive epochs. In other words, the TDCP measurements corresponding to the current epoch are correlated with the TDCP measurements in the next epoch. Considering the correlation between these measurements in the GPS/INS can facilitate estimation of the optimal Kalman gain and final solution. The remainder of this paper is organized as follows. Section 2 describes the theories underlying this study; Section 2.1 shows the detailed composition of the TDCP measurements and the corresponding method of analyzing the positioning accuracy, and Section 2.2 summarizes the equations for the delayed state filter considering noise correlation and the difference between the GPS and INS update rates. Section 3 presents our simulation and experimental results, which confirm the accuracy and reliability improvements attainable by the proposed system. Finally, Section 4 summarizes the main conclusions.

## 2. Theory

### 2.1. Time-Differenced Carrier Phase (TDCP) Measurements

The carrier-phase measurement from the *i*th satellite to the low-cost L1 (1575.42 MHz) single-frequency GPS receiver (expressed in meters) is given by:

(1) $$\phi_{u}^{i}=\left({\bar{r}}^{i}+\delta {\bar{r}}^{i}-{\bar{r}}_{u}\right)\cdot {\bar{e}}_{u}^{i}+B_{u}+T_{u}^{i}-I_{u}^{i}-b^{i}+\varepsilon_{u}^{i}+\lambda N_{u}^{i}$$

The superscript *i* denotes the *i*th satellite, and the subscript *u* denotes the user. $\bar{r},\bar{e},b,B,I,T,\delta \bar{r},N,\lambda$, and ε are the position, line-of-sight vector, satellite clock bias, receiver clock bias, ionospheric delay, tropospheric delay, ephemeris error, integer ambiguity, wavelength of L1, and noise, respectively. 

**Figure 1:**
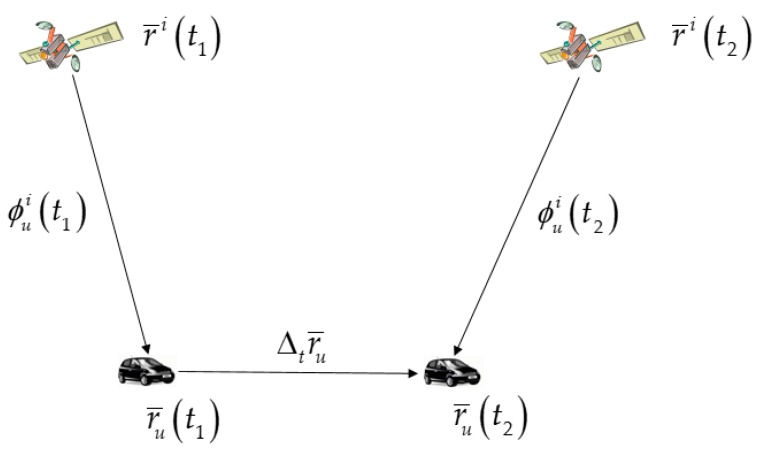
Carrier-phase measurements at time epochs $t_{1}$ and $t_{2}$.

Resolving the integer ambiguity, *N,* in Equation (1) is always difficult, particularly in the nondifferential mode and with a low-cost single-frequency GPS receiver, as considered here. However, *N*, which is a constant, can be eliminated through differencing between two consecutive GPS epochs without requiring resolution. The carrier-phase measurements are shown in Figure 1.

By subtracting the carrier phase measurements at time epochs $t_{1}$ and $t_{2}$, the TDCP measurement can be obtained as follows:

(2) $$\begin{array}{ll} \Delta_{t}\phi_{u}^{i} & =\phi_{u}^{i}\left(t_{2}\right)-\phi_{u}^{i}\left(t_{1}\right)\\ & \;=\left\{{\bar{r}}^{i}\left(t_{2}\right)-{\bar{r}}_{u}\left(t_{2}\right)\right\}\cdot {\bar{e}}_{u}^{i}\left(t_{2}\right)-\left\{{\bar{r}}^{i}\left(t_{1}\right)-{\bar{r}}_{u}\left(t_{1}\right)\right\}\cdot {\bar{e}}_{u}^{i}\left(t_{1}\right)\\ & \;\;+\left\{\delta {\bar{r}}^{i}\left(t_{2}\right)\cdot {\bar{e}}_{u}^{i}\left(t_{2}\right)-\delta {\bar{r}}^{i}\left(t_{1}\right)\cdot {\bar{e}}_{u}^{i}\left(t_{1}\right)\right\}+\Delta_{t}B_{u}+\Delta_{t}T_{u}^{i}-\Delta_{t}I_{u}^{i}-\Delta_{t}b^{i}+\Delta_{t}\varepsilon_{u}^{i}\\ & \;={\bar{r}}^{i}\left(t_{2}\right)\cdot {\bar{e}}_{u}^{i}\left(t_{2}\right)-{\bar{r}}^{i}\left(t_{1}\right)\cdot {\bar{e}}_{u}^{i}\left(t_{1}\right)-\Delta_{t}{\bar{r}}_{u}\cdot {\bar{e}}_{u}^{i}\left(t_{2}\right)-{\bar{r}}_{u}\left(t_{1}\right)\cdot \Delta_{t}{\bar{e}}_{u}^{i}\\ & \;\;+\left\{\delta {\bar{r}}^{i}\left(t_{2}\right)\cdot {\bar{e}}_{u}^{i}\left(t_{2}\right)-\delta {\bar{r}}^{i}\left(t_{1}\right)\cdot {\bar{e}}_{u}^{i}\left(t_{1}\right)\right\}+\Delta_{t}B_{u}+\Delta_{t}T_{u}^{i}-\Delta_{t}I_{u}^{i}-\Delta_{t}b^{i}+\Delta_{t}\varepsilon_{u}^{i} \end{array}$$

where *N* has been eliminated through time differencing. Δ is the time difference operator and signifies the change during period $t_{2}-t_{1}$. In order to calculate the relative position $\Delta_{t}{\bar{r}}_{u}$ using the least squares method, Equation (2) can be modified as follows with *m* visible satellites in matrix form:

(3) $$\begin{array}{l} \Delta_{t}{\bar{r}}_{u}\cdot {\bar{e}}_{u}^{i}\left(t_{2}\right)-\Delta_{t}B_{u}={\bar{r}}^{i}\left(t_{2}\right)\cdot {\bar{e}}_{u}^{i}\left(t_{2}\right)-{\bar{r}}^{i}\left(t_{1}\right)\cdot {\bar{e}}_{u}^{i}\left(t_{1}\right)-{\bar{r}}_{u}\left(t_{1}\right)\cdot \Delta_{t}{\bar{e}}_{u}^{i}-\Delta_{t}\phi_{u}^{i}+\Delta_{t}E_{u}^{i}\\ \left[\begin{array}{cc} {\bar{e}}_{u}^{1}(t_{2}) & -1\\ \vdots & \vdots \\ {\bar{e}}_{u}^{m}(t_{2}) & -1 \end{array}\right]\left(\begin{array}{c} \Delta_{t}{\bar{r}}_{u}\\ \Delta_{t}B_{u} \end{array}\right)=\left[\begin{array}{c} {\bar{r}}^{1}\left(t_{2}\right)\cdot {\bar{e}}_{u}^{1}\left(t_{2}\right)-{\bar{r}}^{1}\left(t_{1}\right)\cdot {\bar{e}}_{u}^{1}\left(t_{1}\right)-{\bar{r}}_{u}\left(t_{1}\right)\cdot \Delta_{t}{\bar{e}}_{u}^{1}-\Delta_{t}\phi_{u}^{1}+\Delta_{t}E_{u}^{1}\\ \vdots \\ {\bar{r}}^{m}\left(t_{2}\right)\cdot {\bar{e}}_{u}^{m}\left(t_{2}\right)-{\bar{r}}^{m}\left(t_{1}\right)\cdot {\bar{e}}_{u}^{m}\left(t_{1}\right)-{\bar{r}}_{u}\left(t_{1}\right)\cdot \Delta_{t}{\bar{e}}_{u}^{m}-\Delta_{t}\phi_{u}^{m}+\Delta_{t}E_{u}^{m} \end{array}\right] \end{array}$$

where $\Delta_{t}E_{u}^{i}=\Delta_{t}T_{u}^{i}-\Delta_{t}I_{u}^{i}-\Delta_{t}b^{i}+\Delta_{t}\varepsilon_{u}^{i}+\left\{\delta {\bar{r}}^{i}\left(t_{2}\right)\cdot {\bar{e}}_{u}^{i}\left(t_{2}\right)-\delta {\bar{r}}^{i}\left(t_{1}\right)\cdot {\bar{e}}_{u}^{i}\left(t_{1}\right)\right\}$ is the total change of the GPS error sources and noise. Equation (3) can also be expressed more simply by using the satellite difference so as not to estimate $\Delta_{t}B_{u}$, as follows:

(4) $$\begin{array}{c} \Delta_{t}{\bar{r}}_{u}\cdot^{i}\nabla^{R}{\bar{e}}_{u}^{}\left(t_{2}\right){=}^{i}\nabla^{R}\bar{r}\left(t_{2}\right)\cdot {\bar{e}}_{u}^{}\left(t_{2}\right){-}^{i}\nabla^{R}\bar{r}\left(t_{1}\right)\cdot {\bar{e}}_{u}^{}\left(t_{1}\right)-{\bar{r}}_{u}\left(t_{1}\right)\cdot^{i}\nabla^{R}\Delta_{t}{\bar{e}}_{u}^{}{-}^{i}\nabla^{R}\Delta_{t}\phi_{u}^{}{+}^{i}\nabla^{R}\Delta_{t}E_{u}^{}\\ \left[\begin{array}{c} {}^{1}\nabla^{R}{\bar{e}}_{u}^{}(t_{2})\\ \vdots \\ {}^{m-1}\nabla^{R}{\bar{e}}_{u}^{}(t_{2}) \end{array}\right]\left(\Delta_{t}{\bar{r}}_{u}\right)\\ =\left[\begin{array}{c} {}^{1}\nabla^{R}\bar{r}\left(t_{2}\right)\cdot {\bar{e}}_{u}^{}\left(t_{2}\right){-}^{1}\nabla^{R}\bar{r}\left(t_{1}\right)\cdot {\bar{e}}_{u}^{}\left(t_{1}\right)-{\bar{r}}_{u}\left(t_{1}\right)\cdot^{1}\nabla^{R}\Delta_{t}{\bar{e}}_{u}^{}{-}^{1}\nabla^{R}\Delta_{t}\phi_{u}^{}{+}^{1}\nabla^{R}\Delta_{t}E_{u}^{}\\ \vdots \\ {}^{m-1}\nabla^{R}\bar{r}\left(t_{2}\right)\cdot {\bar{e}}_{u}^{}\left(t_{2}\right){-}^{m-1}\nabla^{R}\bar{r}\left(t_{1}\right)\cdot {\bar{e}}_{u}^{}\left(t_{1}\right)-{\bar{r}}_{u}\left(t_{1}\right)\cdot^{m-1}\nabla^{R}\Delta_{t}{\bar{e}}_{u}^{}{-}^{m-1}\nabla^{R}\Delta_{t}\phi_{u}^{}{+}^{m-1}\nabla^{R}\Delta_{t}E_{u}^{} \end{array}\right] \end{array}$$

The superscript *R* denotes the reference satellite, which is typically defined as the highest elevated satellite among all of the satellites because it could have the smallest error factors and noise in the satellite difference represented by ∇. 

Equation (4) can be expressed in the Hx=z format, and $\Delta_{t}{\bar{r}}_{u}$ can be determined as follows:

(5) $$\Delta_{t}{\bar{r}}_{u}={\left(H^{T}H\right)}^{-1}H^{T}z$$

To confirm the positioning accuracy by using TDCP measurements, a static simulation and an actual experiment were conducted. In the simulation, most of the GPS error sources were generated based on models such as the wide area augmentation system (WAAS) model [17] for tropospheric delay and the exponential model [18] for noise. In addition, the ephemeris error and satellite clock bias were generated based on comparative analysis of GPS broadcast ephemeris data and standard product #3 (SP3) data as precise ephemeris. The ionospheric delay was generated by using actual post-processing precise data such as ionosphere exchange-format data from the Jet Propulsion Laboratory [19], and the integer ambiguity of each satellite and receiver clock bias were assumed to be random integers not affecting the TDCP-based positioning because of the time and satellite difference in Equations (2) and (4). In the actual static test, a Trimble NetR9 GPS receiver and Trimble Zephyr 2 Geodetic Antenna installed at Seoul National University were utilized to acquire satellite signals. The relative positions obtained, as shown in Equation (5), were accumulated from the initial position. 

**Figure 2:**
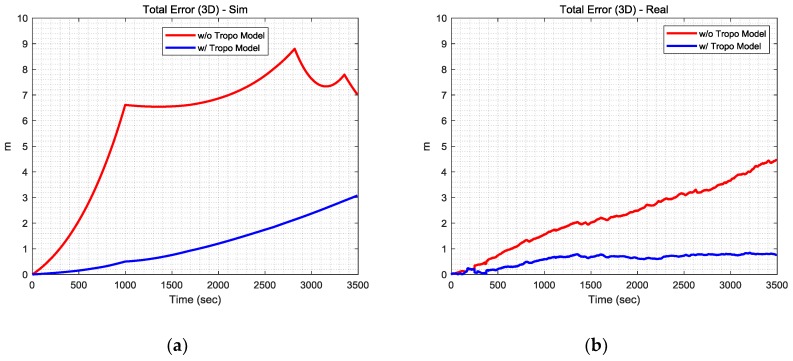
(**a**) Static simulation results; (**b**) static experimental results.

Figure 2 shows the TDCP-based positioning results of the static simulation and experimental test. Because tropospheric delay causes most of the positioning errors in quiet ionospheric conditions, it should be eliminated in order to obtain precise positioning information by using TDCP measurements. Furthermore, it can mostly be estimated using the model. Therefore, the simulation and experimental results were compared with those of the tropospheric model. The Saastamoinen tropospheric model [20] was employed as a model different from that used to generate the simulation data. As shown in Figure 2, the positioning error tends to increase significantly over time without the tropospheric model. On the other hand, there are significant reductions in the tendency of the positioning error to increase over time with the model. Closer inspection reveals a three-dimensional positioning error of approximately 3 m per hour in the simulation results and approximately 1 m per hour in the real test results. Furthermore, it is possible to obtain more precise navigation solutions if differential information such as that from a differential GPS or satellite-based augmentation system can be used to reduce the temporal variations of other error factors [21]. 

### 2.2. TDCP-Based Global Positioning System/Inertial Navigation System (GPS/INS).

Before presenting the derivation of a measurement matrix to process the TDCP measurements in the KF, the derivations of the basic KF equations are summarized. Generally, systems such as autonomous vehicles and drones have the properties of nonlinear systems. Thus, this paper focuses on an extended KF (EKF) for a non-linear system, and the process and measurement models of a basic EKF can be defined as follows:

(6) $$\begin{array}{l} \delta x_{k+1}=\Phi_{k}\delta x_{k}+w_{k}\\ \delta z_{k}=H_{k}\delta x_{k}+v_{k} \end{array}$$

The subscripts *k* + 1 and *k* denote the *k*+1th and *k*th time epochs, respectively; Φ and *H* are the state transition and observation matrix, respectively; and δ∗ denotes the residual error of variable ∗, which means the difference between the estimated value $\hat{*}$ and the true value ${*}_{true}$. It can be defined as follows:

(7) $$\delta *=\hat{*}-{*}_{true}$$

The process noise *w* and measurement noise *v* are usually not cross-correlated and have the following characteristics:

(8) $$\begin{array}{l} E\left[w_{k}w_{k}^{T}\right]=\{\begin{array}{c} Q_{k},\;\;i=k\\ 0,\;\;i\neq k \end{array}\\ E\left[v_{k}v_{k}^{T}\right]=\{\begin{array}{c} R_{k},\;\;i=k\\ 0,\;\;i\neq k \end{array} \end{array}$$

However, if there is a cross-correlation between $w_{k-1}$ and $v_{k}$, their cross-correlation should be considered in order to obtain the optimal Kalman gain *K* that determines the EKF performance, which can be expressed as follows:

(9) $$E\left[w_{k-1}v_{k}^{T}\right]=C_{k}$$

The TDCP-based KF methods described in Section 1 cannot provide optimal navigation solutions because the filters are general KFs without consideration of the cross-correlation process and measurement noise. Next, the process of constructing a filter considering cross-correlation is explained; it starts with the following measurement update equation:

(10) $$\delta {\hat{x}}_{k}^{+}=\delta {\hat{x}}_{k}^{-}+K_{k}\left(\delta z_{k}-H_{k}\delta {\hat{x}}_{k}^{-}\right)$$

where the superscripts – and + denote the states before and after measurement update. The measurement model $\delta z_{k}$ of the basic EKF is typically constructed based on information calculated using the GPS. (The construction of the measurement model using the TDCP measurements will be explained in greater detail later.) 

Next, the estimation error or residual error can be defined as follows:

(11) $$\begin{array}{l} e_{k}=\delta {\hat{x}}_{k}^{+}-\delta x_{k}\\ \;=\delta {\hat{x}}_{k}^{-}+K_{k}\left(\delta z_{k}-H_{k}\delta {\hat{x}}_{k}^{-}\right)-\delta x_{k}\\ \;=\delta {\hat{x}}_{k}^{-}-\delta x_{k}+K_{k}\left(H_{k}\delta x_{k}+v_{k}-H_{k}\delta {\hat{x}}_{k}^{-}\right)\\ \;=\left(I-K_{k}H_{k}\right)e_{k}^{-}+K_{k}v_{k} \end{array}$$

By using Equation (11), the covariance matrix $P_{k}^{+}$ can be derived as follows:

(12) $$\begin{array}{ll} P_{k}^{+} & =E\left[e_{k}e_{k}{}^{T}\right]\\ & =E\left\{\left[\left(I-K_{k}H_{k}\right)e_{k}^{-}+K_{k}v_{k}\right]{\left[\left(I-K_{k}H_{k}\right)e_{k}^{-}+K_{k}v_{k}\right]}^{T}\right\} \end{array}$$

Herein, it is necessary to identify the relationship between $e_{k}^{-}$ and $v_{k}$ in order to expand Equation (12), which can be expressed as follows:

(13) $$\begin{array}{ll} E\left[e_{k}^{-}v_{k}^{T}\right] & =E\left[\left(\delta {\hat{x}}_{k}^{-}-\delta x_{k}\right)v_{k}^{T}\right]\\ & \;=E\left[\left(\Phi_{k-1}\delta {\hat{x}}_{k-1}-\Phi_{k-1}\delta x_{k-1}-w_{k-1}\right)v_{k}^{T}\right]\\ & \;=-E\left[w_{k-1}v_{k}^{T}\right]=-C_{k} \end{array}$$

Note that $v_{k}$ will not be correlated with either $\delta x_{k-1}$ or $\delta {\hat{x}}_{k-1}$ because it is of white characteristics. Therefore, Equation (12) can be expanded as follows:

(14) $$\begin{array}{ll} P_{k}^{+} & =\left(I-K_{k}H_{k}\right)P_{k}^{-}{\left(I-K_{k}H_{k}\right)}^{T}+K_{k}R_{k}K_{k}^{T}\\ & \;-\left(I-K_{k}H_{k}\right)C_{k}K_{k}^{T}-K_{k}C_{k}^{T}{\left(I-K_{k}H_{k}\right)}^{T} \end{array}$$

Equation (14) is a perfectly general expression for the error covariance and is valid for any Kalman gain $K_{k}$. The last two terms in Equation (14) are related to cross-correlation, which was not considered in the studies outlined in Section 1. The optimal Kalman gain can be obtained by differentiating trace $P_{k}^{+}$ with respect to $K_{k}$ and setting the result equal to zero, as follows:

(15) $$K_{k}=\left(P_{k}^{-}H_{k}^{T}+C_{k}\right){\left[H_{k}P_{k}^{-}H_{k}^{T}+R_{k}+H_{k}C_{k}+C_{k}^{T}H_{k}^{T}\right]}^{-1}$$

**Figure 3:**
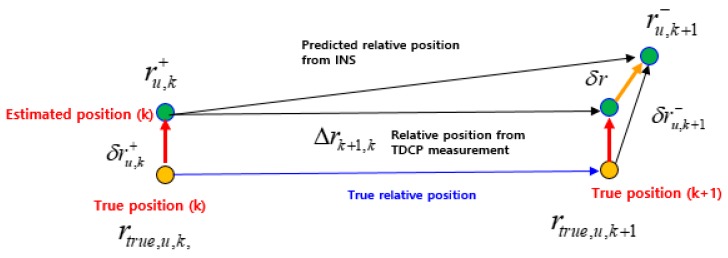
Relationships among all position vectors in two consecutive epochs.

Next, we look at why the cross-correlation should be considered when using TDCP measurements. The relative position $\Delta_{t}{\bar{r}}_{u}$ when using TDCP measurements can be identified from Equation (5) and can be expressed in two consecutive epochs as $\Delta r_{k+1,k}$. Figure 3 shows the relationships among all position vectors in two consecutive epochs, where $r_{true,u,k}$ and $r_{true,u,k+1}$ are defined as the true positions, and $r_{u,k}^{+}$ and $\delta r_{u,k}^{+}$ are defined as the position estimated by the EKF and its error, respectively, at time *k*. The position $r_{u,k+1}^{-}$ in the next epoch estimated by INS-based time propagation has an error $\delta r_{u,k+1}^{-}$ relative to $r_{true,u,k+1}$. Therefore, it is necessary to compensate for the error based on the relative position $\Delta r_{k+1,k}$ from the TDCP measurements, assuming that the relative position based on the TDCP measurements is equal to the true relative position. 

As shown in Figure 3, δr can be expressed in two ways. Firstly, it can be expressed through the vector relation represented by the small triangle on the right, as follows:

(16) $$\delta r=\delta r_{u,k+1}^{-}-\delta r_{u,k}^{+}$$

Secondly, it can be expressed through the vector relation represented by the upper triangle, as follows:

(17) $$\delta r=r_{u,k+1}^{-}-r_{u,k}^{+}-\Delta r_{k+1,k}$$

The final relation can be expressed by integrating Equations (16) and (17), as follows:

(18) $$r_{u,k+1}^{-}-r_{u,k}^{+}-\Delta r_{k+1,k}=\delta r_{u,k+1}^{-}-\delta r_{u,k}^{+}$$

On the left side of Equation (18), $r_{u,k+1}^{-}-r_{u,k}^{+}$ is the relative position estimated by the INS and $\Delta r_{k+1,k}$ is the relative position from TDCP-based positioning. Both relative positions correspond to measurement $\delta z_{k+1}$ of EKF. On the right side of Equation (18), $\delta r_{u,k+1}^{-}$ and $\delta r_{u,k}^{+}$ correspond to the states of the EKF. In other words, Equation (18) represents the measurement equation of the EKF. Because it contains current states as well as past states, it is contrary to the assumption of a normal EKF and requires a different EKF. In this paper, we introduce the concept of a delayed-state KF and its modification for an EKF [16,22].

The measurement consists of information from two consecutive epochs, such as the relative position obtained through TDCP measurement, and can be expressed as:

(19) $$\delta z_{k+1}=H_{k+1}\delta x_{k+1}+J_{k+1}\delta x_{k}+v_{k+1}$$

where J is another observation matrix, which is similar to H. The definitions of J and H will be provided later.

From Equation (6), it is possible to consider backward time propagation, as follows:

(20) $$\delta x_{k}=\Phi_{k}^{-1}\delta x_{k+1}-\Phi_{k}^{-1}w_{k}$$

Equation (20) can be substituted into Equation (19), and a new measurement equation derived for TDCP measurements as follows:

(21) $$\begin{array}{l} \delta z_{k+1}=H_{k+1}\delta x_{k+1}+J_{k+1}\left(\Phi_{k}^{-1}\delta x_{k+1}-\Phi_{k}^{-1}w_{k}\right)+v_{k+1}\\ \;\;=\left(H_{k+1}+J_{k+1}\Phi_{k}^{-1}\right)\delta x_{k+1}+\left(-J_{k+1}\Phi_{k}^{-1}w_{k}+v_{k+1}\right)\\ \;\;≜{H^{\prime }}_{k+1}\delta x_{k+1}+{v^{\prime }}_{k+1} \end{array}$$

As shown in Equation (21), the new quantities ${H^{\prime }}_{k+1}$ and ${v^{\prime }}_{k+1}$ are obtained and it is necessary to derive new covariance matrices associated with them, as follows:

(22) $$\begin{array}{l} {R^{\prime }}_{k+1}=E\left[{v^{\prime }}_{k+1}{v^{\prime }}_{k+1}{}^{T}\right]\\ \;=E\left[\left(-J_{k+1}\Phi_{k}^{-1}w_{k}+v_{k+1}\right){\left(-J_{k+1}\Phi_{k}^{-1}w_{k}+v_{k+1}\right)}^{T}\right]\\ \;=J_{k+1}\Phi_{k}^{-1}Q_{k}\Phi_{k}^{-1T}J_{k+1}^{T}+R_{k} \end{array}$$

(23) $$\begin{array}{l} C_{k+1}=E\left[w_{k}{v^{\prime }}_{k+1}{}^{T}\right]\\ \;=E\left[w_{k}{\left(-J_{k+1}\Phi_{k}^{-1}w_{k}+v_{k+1}\right)}^{T}\right]\\ \;=-Q_{k}\Phi_{k}^{-1T}J_{k+1}^{T} \end{array}$$

Thus, the optimal Kalman gain and solution considering the cross-correlation by using the TDCP-based relative position can be obtained by applying the newly derived ${H^{\prime }}_{k+1}$, ${R^{\prime }}_{k+1}$, and $C_{k+1}$ to the measurement update equations, such as Equations (10), (14), and (15). The time update, which is the same as that for a normal EKF, can be summarized as follows:

(24) $$\begin{array}{l} \delta {\hat{x}}_{k+1}^{-}=\Phi_{k}\delta {\hat{x}}_{k}^{+}\\ P_{k+1}^{-}=\Phi_{k}P_{k}^{+}\Phi_{k}^{T}+Q_{k} \end{array}$$

However, actual GPS/INS integration based on the above equations is difficult. The equations can only be used if the GPS and INS have the same sample rate. Usually, a GPS has a 1 Hz sample rate and an INS has a 100 Hz sample rate. Thus, the difference between the sample rates should be considered, and it is difficult to find reports that provide equations considering this difference. Therefore, we present the time and measurement update equations considering the sample rates. As the process could be too complicated if the INS sample rate is 100 Hz, we provide the derivation for the 3 Hz case as an example and extend it to the 100 Hz case. 

**Figure 4:**
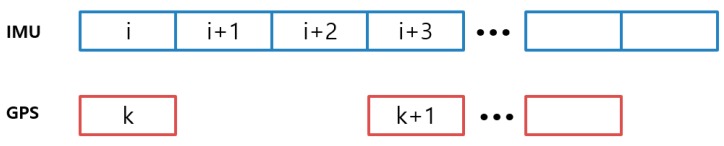
Difference between global positioning system (GPS) (1 Hz) and inertial navigation system (INS) (3 Hz) data output.

Figure 4 shows the difference between the GPS and INS data output. The measurement equation at *k* + 1 can be expressed as follows:

(25) $$\begin{array}{l} \delta z_{k+1}=H\delta x_{k+1}+J\delta x_{k}+v_{k+1}\\ \;\;=H\delta x_{i+3}+J\delta x_{i}+v_{k+1} \end{array}$$

where it is assumed that the observation matrices *H* and *J* are time-invariant. In this study, as the LC was constructed using only the relative positions based on the TDCP measurements, there is no problem with the above assumption. The forward time update equations can be organized as follows:

(26) $$\begin{array}{l} \delta x_{i+1}=\Phi_{i}\delta x_{i}+w_{i}\\ \delta x_{i+2}=\Phi_{i+1}\Phi_{i}\delta x_{i}+\Phi_{i+1}w_{i}+w_{i+1}\\ \delta x_{i+3}=\Phi_{i+2}\Phi_{i+1}\Phi_{i}\delta x_{i}+\Phi_{i+2}\Phi_{i+1}w_{i}+\Phi_{i+2}w_{i+1}+w_{i+2} \end{array}$$

In order to construct the *k* + 1 or *i* + 3 states corresponding to the current in Equation (25), Equation (26) should be rearranged as a backward time update equation as follows:

(27) $$\begin{array}{l} \delta x_{i+2}=\Phi_{i+2}^{-1}\delta x_{i+3}-\Phi_{i+2}^{-1}w_{i+2}\\ \delta x_{i+1}=\Phi_{i+1}^{-1}\Phi_{i+2}^{-1}\delta x_{i+3}-\Phi_{i+1}^{-1}\Phi_{i+2}^{-1}w_{i+2}-\Phi_{i+1}^{-1}w_{i+1}\\ \delta x_{i}=\Phi_{i}^{-1}\Phi_{i+1}^{-1}\Phi_{i+2}^{-1}\delta x_{i+3}-\Phi_{i}^{-1}\Phi_{i+1}^{-1}\Phi_{i+2}^{-1}w_{i+2}-\Phi_{i}^{-1}\Phi_{i+1}^{-1}w_{i+1}-\Phi_{i}^{-1}w_{i} \end{array}$$

Substitution of Equation (27) into Equation (25) yields the current states, as follows:

(28) $$\begin{array}{l} \delta z_{k+1}=H\delta x_{i+3}+J\delta x_{i}+v_{k+1}\\ \;\;=H\delta x_{i+3}+J\left(\Phi_{i}^{-1}\Phi_{i+1}^{-1}\Phi_{i+2}^{-1}\delta x_{i+3}-\Phi_{i}^{-1}\Phi_{i+1}^{-1}\Phi_{i+2}^{-1}w_{i+2}-\Phi_{i}^{-1}\Phi_{i+1}^{-1}w_{i+1}-\Phi_{i}^{-1}w_{i}\right)+v_{k+1}\\ \;\;=\left(H+J\Phi_{i}^{-1}\Phi_{i+1}^{-1}\Phi_{i+2}^{-1}\delta x_{i+3}\right)\delta x_{i+3}+J\left(-\Phi_{i}^{-1}\Phi_{i+1}^{-1}\Phi_{i+2}^{-1}w_{i+2}-\Phi_{i}^{-1}\Phi_{i+1}^{-1}w_{i+1}-\Phi_{i}^{-1}w_{i}\right)+v_{k+1} \end{array}$$

${H^{\prime }}_{k+1}$, ${R^{\prime }}_{k+1}$, and $C_{k+1}$ can be obtained through the same logic as was applied to obtain Equations (21)–(23), as follows:

(29) $$\begin{array}{l} {H^{\prime }}_{k+1}=H+J\Phi_{i+2,i}^{-1}\delta x_{i+3}\\ {R^{\prime }}_{k+1}=E\left[AA^{T}\right]=J\Phi_{i+2,i}^{-1}Q{\left(\Phi_{i+2,i}^{-1}\right)}^{T}J^{T}+J\Phi_{i+1,i}^{-1}Q{\left(\Phi_{i+1,i}^{-1}\right)}^{T}J^{T}+J\Phi_{i,i}^{-1}Q{\left(\Phi_{i,i}^{-1}\right)}^{T}J^{T}+R\\ C_{k+1}=E\left[BB^{T}\right]=-Q{\left(\Phi_{i+2,i}^{-1}\right)}^{T}J^{T}-\Phi_{i+2}Q{\left(\Phi_{i+1,i}^{-1}\right)}^{T}J^{T}-\Phi_{i+2}\Phi_{i+1}Q{\left(\Phi_{i,i}^{-1}\right)}^{T}J^{T} \end{array}$$

where $A=J\left(-\Phi_{i}^{-1}\Phi_{i+1}^{-1}\Phi_{i+2}^{-1}w_{i+2}-\Phi_{i}^{-1}\Phi_{i+1}^{-1}w_{i+1}-\Phi_{i}^{-1}w_{i}\right)+v_{k+1}$, $B=\Phi_{i+2}\Phi_{i+1}w_{i}+\Phi_{i+2}w_{i+1}+w_{i+2}$, and $\Phi_{i+j,i}^{-1}=\Phi_{i}^{-1}\Phi_{i+1}^{-1}\cdots \Phi_{i+j}^{-1}$. In addition, it is assumed that *Q* and *R* are also time-invariant. Equation (29) can be extended to the 100 Hz case as follows:

(30) $$\begin{array}{l} {H^{\prime }}_{k+1}=H+J\Phi_{i+99,i}^{-1}\delta x_{i+100}\\ {R^{\prime }}_{k+1}=R+\sum_{j=0}^{99}J\Phi_{i+j,i}^{-1}Q{\left(\Phi_{i+j,i}^{-1}\right)}^{T}J^{T}\\ C_{k+1}=-\sum_{j=0}^{99}\Phi_{i+99,i}\Phi_{i+j,i}^{-1}Q{\left(\Phi_{i+j,i}^{-1}\right)}^{T}J^{T} \end{array}$$

where $\Phi_{i+j,i}^{}=\Phi_{i+j}^{}\cdots \Phi_{i+1}^{}\Phi_{i}^{}$. Although this quantity can be approximated using various methods [13,14,15], in this approach, it is computed by simple multiplication by definition as the system matrices at each time update epoch are calculated and can be stored.

**Figure 5:**
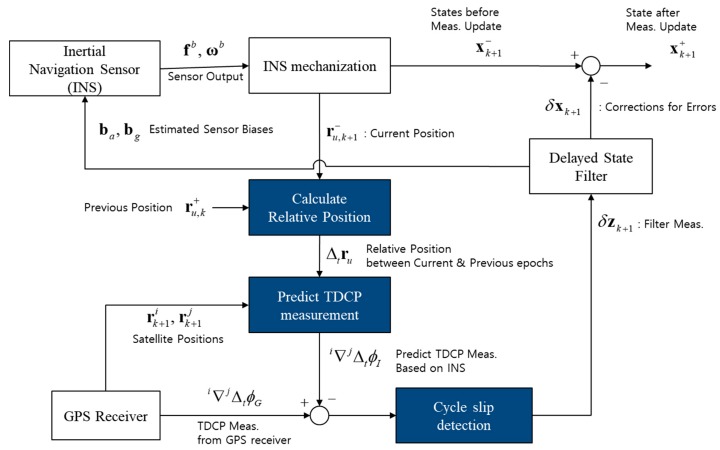
Flowchart of time-differenced carrier phase (TDCP)-based integrated GPS/INS.

Figure 5 shows the flowchart of TDCP-based integrated GPS/INS. In this system, a delayed state filter, as described in the above equations, is used for the INS error dynamics and INS error model. The state vector of the filter consists of 15 elements, which are as follows:

(31) $$x=\left[\begin{array}{ccccc} \delta r & \delta v & \varepsilon & \delta b_{a} & \delta b_{g} \end{array}\right]$$

where δr are the position errors in longitude, latitude, and height; δv are the velocity errors towards the north, east, and down; ε are the attitude errors expressed in Ψ; and δb are the bias errors of the INS. The subscripts *a* and *g* denote the accelerometer and gyroscope, respectively. The equations for the inertial measurement unit (IMU) error can be found in [1,5,15]. In this study, as the TDCP-based relative position was used as the measurement of the LC filter, the observation matrices were defined as follows:

(32) $$\begin{array}{l} H=\left[\begin{array}{cc} I_{3\times 3} & 0_{3\times 15} \end{array}\right]\\ J=-H \end{array}$$

When each TDCP measurement is used as the measurement of the TC filter, the observation matrices are composed of line of sight vectors, which can be found in [10,14].

The TDCP measurements used in this study may cause large positioning errors owing to cycle slip of the carrier phase measurement. Thus, an algorithm to deal with the cycle slip is necessary. To this end, it is assumed that all cycle slips can be detected via the method described in [23]. 

## 3. Simulation and Experimental Results

Simulation and real experiments of a dynamic user were conducted to confirm the performance improvement of the delayed state filter that considers the noise correlation as in Equation (30). To confirm the improvement, a conventional filter was constructed as a control that did not consider the noise correlation and consisted of the same observability matrix and different covariance matrices, as shown in Table 1. All of the filters were programmed in MATLAB (R2018a) and run on a computer with an Intel Core i7-6700K central processing unit (4.0 GHz) and 16 GB of random access memory.

### 3.1. Preliminary Test

**Figure 6:**
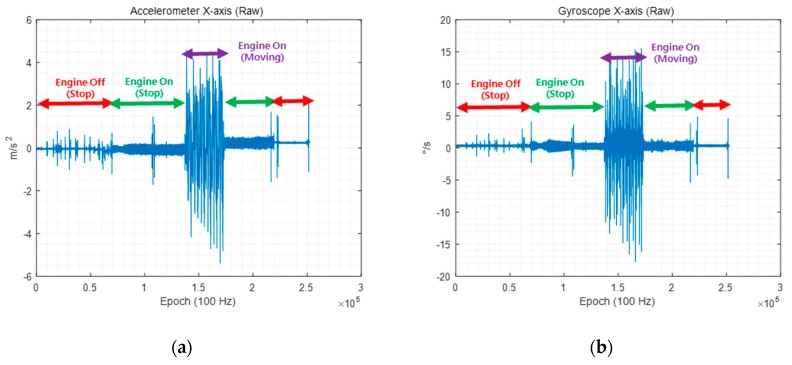
(**a**) x-axis accelerometer output; (**b**) x-axis gyroscope output.

Before comparing the performances of the conventional and delayed state filters in the simulation and experiments, a preliminary test was conducted to check the noise level measured by the INS on a land vehicle because proper filter design must accurately reflect the noise level. The INS has its own noise level, which is generally determined by the user based on the sensor documentation. However, the noise level measured by the INS increases with the dynamics of the land vehicle and environmental factors such as vibrations due to the engine and road surface conditions [24,25,26]. Figure 6 shows the x-axis outputs of the INS sensor embedded in the Ublox M8L receiver, which consists of a low-cost GPS receiver, three-axis accelerometers, and three-axis gyroscopes. A land vehicle with an INS installed moved in the following order: engine off while stopping, engine on while stopping, engine on while moving, engine on while stopping, and engine off while stopping.

As shown in Figure 6, the noise level in the INS becomes very large when the engine is turned on. The noise levels of the INS in each situation are summarized in Table 2. 

As shown in Table 2, the noise levels of the x- and y-axis accelerometers are 10 times higher and that of the z-axis accelerometer is four times higher when the engine is turned on and off. In addition, the noise levels of the x- and y-axis gyroscopes are five times higher when the engine is turned on and off. In this study, noise analysis was not performed for the section in which the vehicle moved. However, this state can be expected to have more noise than when the vehicle is stationary owing to various factors such as the road surface conditions. Based on the above noise analysis results, we set up scenarios with the noise level constraint equal to the IMU sensor specifications as well as 2.5 and 5 times this noise level for the simulation and actual experiments and analyzed the results.

### 3.2. Simulation

#### 3.2.1. Simulation Environment

**Figure 7:**
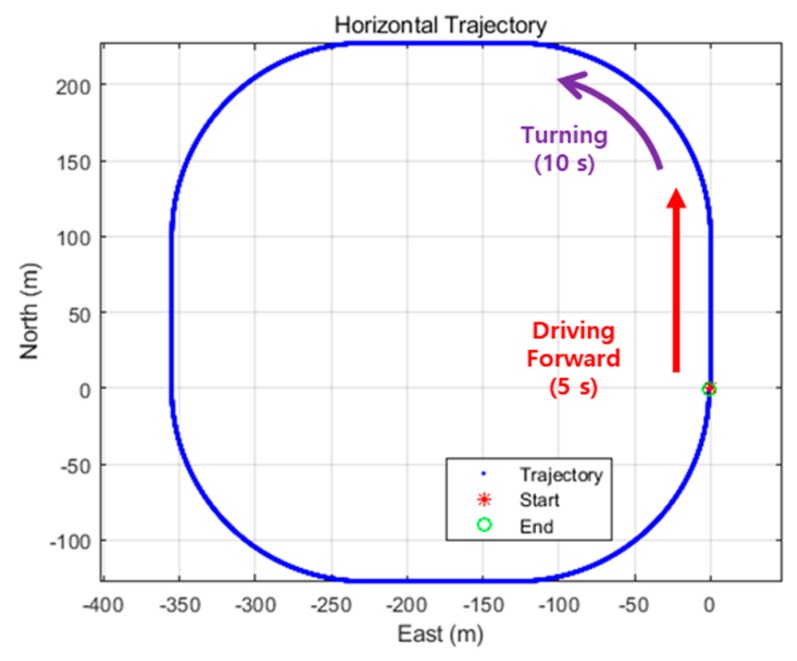
Generated user trajectory (simulation).

To verify the proposed algorithm, 900 s simulation data were generated for a trajectory with driving forward for 5 s and turning 90° for 10 s at a constant speed of 20 m/s, as shown in Figure 7. The speed may not significantly affect the results. The GPS simulation data were generated with all GPS errors and the receiver noise of the low-cost GPS receiver, except for the tropospheric delay, which can mostly be removed using the model. The GPS output rate was set to 1 Hz. The INS data were generated for three cases. The data for the first case were generated according to the specifications of an ADIS16405IMU [27], which is of industrial MEMS grade [28]. The other two cases were assumed to have noise levels 2.5 and 5 times the sensor specifications, taking into account the environmental factors analyzed in the abovementioned preliminary test. The INS output rate was set to 100 Hz. As the user starts from an already known point, the initial position standard deviation is set to zero in the initial state covariance matrix ($P_{0}$).

#### 3.2.2. Simulation Results

**Figure 8:**
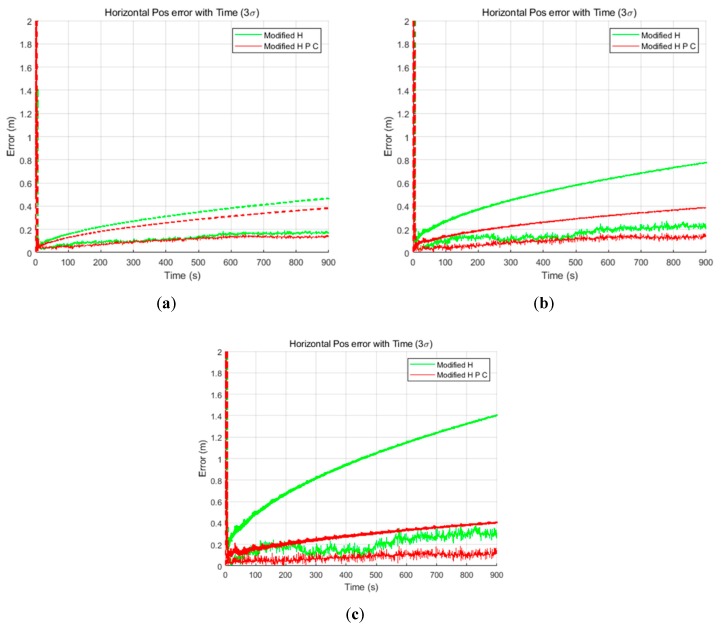
Horizontal results of the (**a**) inertial measurement unit (IMU) sensor specification case, (**b**) 2.5× case, and (**c**) 5× case.

The TDCP-based GPS/INS delayed state filter was implemented using generated GPS and INS data. Furthermore, in order to compare the performance, a conventional filter was also implemented. Both filters were constructed as LC EKFs, and the relative positions obtained from the TDCP measurements were used as the filter measurements. Three simulations were performed, one for each noise level. The process noise of the filter was tuned assuming that the noise level was known, as in the common KF tuning method. Figure 8 shows the horizontal results of the three cases in order. In this paper, the horizontal results are summarized because the focus is on improving the performance of the navigation system for land vehicles. In Figure 8, the dashed line indicates the 3σ value estimated by the filter and the solid line indicates the positioning error. Further, the green and red curves represent the results obtained using the conventional and delayed state filters, respectively. As shown in Figure 8, the position errors and the 3σ values estimated by the delayed state filter are smaller than those estimated by the conventional filter. These results can be explained by the fact that there is a difference in the estimation performance between the Kalman gain and covariance optimally depending on the consideration of noise correlation generated using the TDCP measurements. 

**Figure 9:**
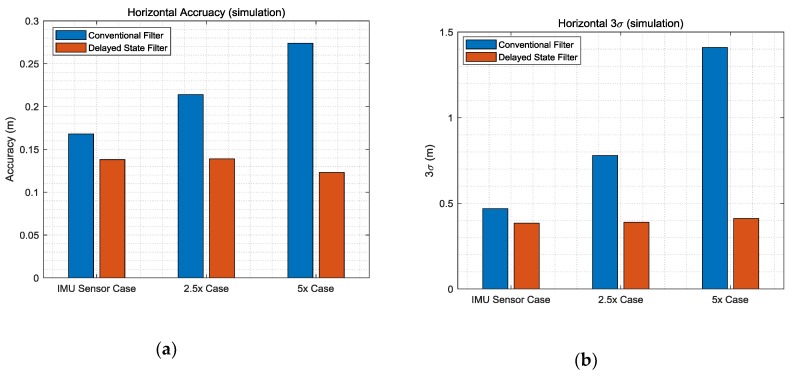
(**a**) Horizontal accuracy; (**b**) horizontal 3σ.

Table 3 lists the detailed numerical results obtained at the final time. It is meaningful to check the final values because the relative values gradually accumulate and have absolute values for the two filters using TDCP measurements. The 3σ results reveal performance improvements of 20%, 50%, and 70% in the IMU sensor case, 2.5× case, and 5× case, respectively. Figure 9 shows the horizontal results of Table 3. In the case of the conventional filter, in order to reflect the sensor noise level, filter tuning was performed to increase the process noise. As a result, it can be seen that the larger the noise, the larger the σ accumulation over time. On the other hand, in the case of the delayed state filter, as the influence of the σ of the previously estimated states can be effectively reduced considering the noise correlation, as shown in Equations (22) and (23), the performance can be improved and the results are similar for all three simulation environments. Furthermore, to analyze the computational load of the proposed filter, the average computation times for measurement update with the conventional filter and delayed state filter methods were computed. Table 4 lists the calculation results.

The computation time is increased by 20% in the delayed state filter case. However, the time is very short for both filters. Therefore, we can conclude that the computational load of the proposed algorithm is permissible. 

#### 3.2.3. Monte Carlo Simulation Results

**Figure 10:**
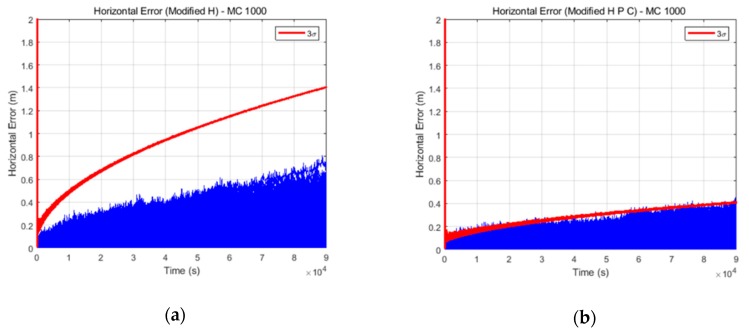
Horizontal results obtained from the (**a**) conventional and (**b**) delayed state filter Monte Carlo simulations.

A Monte Carlo simulation was performed for further verification of the proposed algorithm. The environment of the previous simulation was mainly used. In the Monte Carlo simulation, several assumptions were applied in order to exclude error factors not following the Gaussian distribution because the EKF in essence assumes that all of the error components follow a Gaussian distribution. If the error factors not following the Gaussian distribution are included, an accurate Monte Carlo test would not be performed. One of the several assumptions is that, in the case of INS data, constant bias components were excluded. In the case of GPS data, all GPS error factors were excluded except for GPS receiver noise. Then, the relative positions determined by the TDCP measurements followed Gaussian distributions. The 5× case INS data were used to identify the performance difference between two filters clearly. In the Monte Carlo simulation, 32 different GPS and INS data were generated and a total of 1024 tests were performed. Figure 10 shows the horizontal results of the Monte Carlo simulation. Figure 10a,b present the results obtained using the conventional filter and delayed state filter, respectively. The blue lines indicate the positioning errors of the 1024 tests and the red lines indicate the 3σ values estimated by the filter. As shown in Figure 10, the position errors and 3σ values estimated using the delayed state filter are smaller than those estimated using the conventional filter. Furthermore, it can be seen that the conventional filter results are over-bound, whereas the delayed state filter results are optimally bounded by 3σ.

Table 5 lists the detailed numerical results corresponding to the final time. For accuracy, the RMS of the final position errors of 1024 tests were calculated and are summarized herein. These results confirm that the proposed filter considering the noise correlation has much better performance than the conventional filter.

### 3.3. Experiment

#### 3.3.1. Experimental Environment

**Figure 11:**
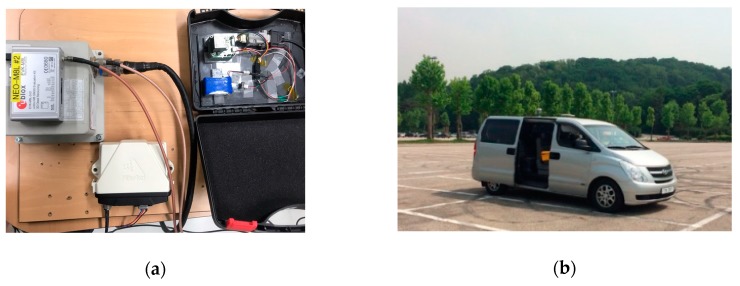
(**a**) Hardware setup; (**b**) land vehicle.

For a more practical test of the proposed algorithm, we conducted a vehicle-based dynamic experiment. This dynamic experiment was performed for 15 min at the Seoul Grand Park parking lot on 16 December 2015. Figure 11a,b show the hardware setup and the land vehicle used in the experiment, respectively. A Novatel FlexPak-G2 was selected as the single-frequency, low-cost GPS receiver, and an ADIS16405IMU was used as the INS. The GPS output rate was set to 1 Hz, and the INS output rate was set to 100 Hz. In the experiment, tropospheric delays were mostly removed through the WAAS model and by using GPS measurements.

**Figure 12:**
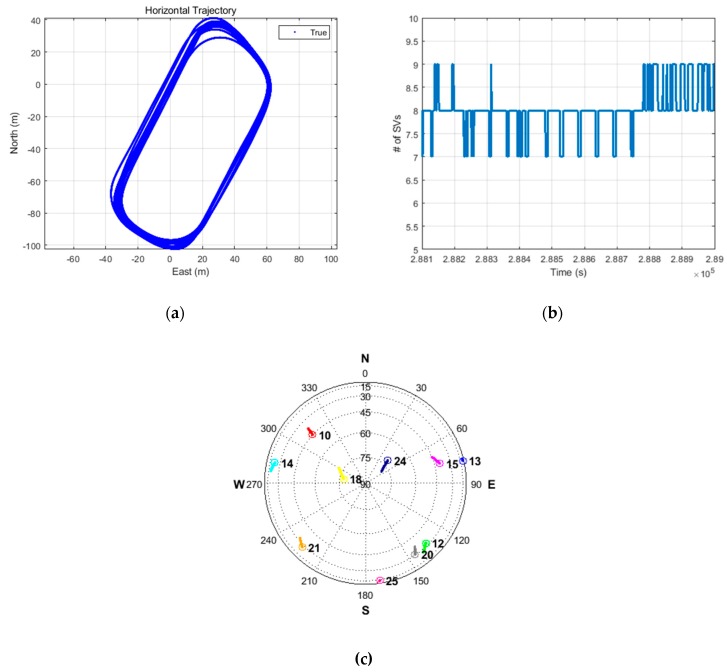
(**a**) Tracked trajectory; (**b**) number of visible satellites; (**c**) sky plot.

Figure 12a–c show the tracked trajectory, number of satellites during the experiment, and sky plot during the experiment, respectively.

#### 3.3.2. Experimental Results

**Figure 13:**
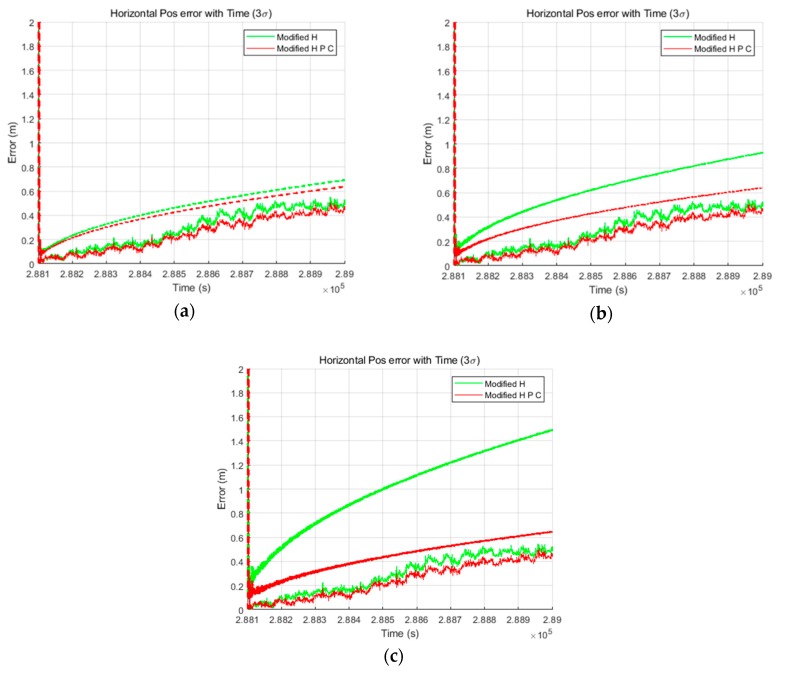
Horizontal results of the (**a**) IMU sensor specification case, (**b**) 2.5× case, and (**c**) 5× case.

All of the methods were the same as those in the simulation except that the data were actually collected. In the experimental process, the same three process noise settings that were assumed in the simulation were employed. This setup was used because the noise level measured by the INS is not known precisely, but it is clear that the noise is larger than the sensor specification, as shown in the preliminary test. Thus, the process noise of the filter was tuned the same as in the simulation. Figure 13 shows the horizontal results obtained in the three cases and confirms that the performance of the delayed state filter was much better than that of the conventional filter in the actual experiment. In addition, it is evident that the error is flowing in one direction owing to the variations of the GPS error sources in the TDCP measurements, but it is clear that the estimation results are good because the position errors are within 3σ values estimated by both filters. However, the estimated σ values of the conventional filter are over-bound, which means that the time taken to ensure lane classification quality for land vehicles is not sufficient.

**Figure 14:**
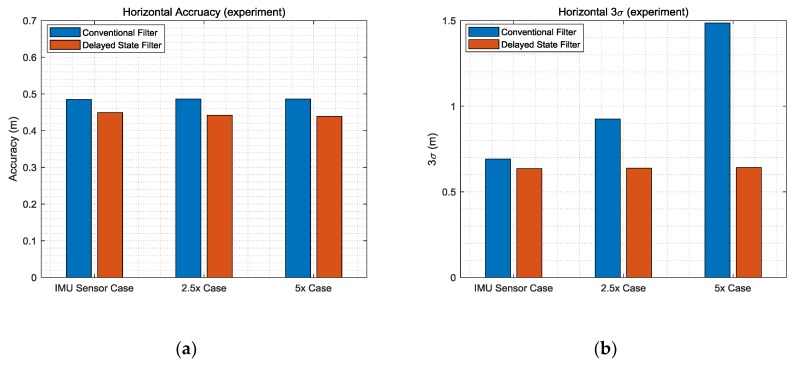
(**a**) Horizontal accuracy; (**b**) horizontal 3σ.

Table 6 lists the detailed numerical results, which correspond to the final time. The 3σ results demonstrate the performance improvement by 10%, 30%, and 60% in the IMU sensor specification case, 2.5× case, and 5× case, respectively. Figure 14 shows the horizontal results of Table 6. The reason that the improvements in accuracy are less than those in the simulation is that the maneuvers of the user as well as the road environment, which cause noise, were better than in the simulation environment. As a result of the use of lower-grade IMU sensors and poor road environment, the improvement of the performance of the delayed state filter could be prominent.

## 4. Conclusions

In this study, we designed a TDCP-based integrated GPS/INS navigation system that appropriately considers noise correlation. Because the TDCP contains current and previous information that violate the format of the usual KF, approximations have been used to design new measurement equations in most studies. However, the noise correlations that occur when using TDCP measurements have not been considered. Thus, we proposed an algorithm that considers the noise correlations for optimal estimation performance and reliability analysis, such as covariance bounding. In the filter using the TDCP measurements, the process noise *Q* at time update and measurement noise *R* at measurement update are accumulated, enabling filter covariance estimation. If the noise correlation is not considered, the covariance of the previous state has a significant influence on the current state estimation at the measurement update, and a non-optimal large covariance is obtained. On the other hand, in the proposed algorithm considering the noise correlations, the influence of the covariance of the previous state can be effectively reduced, and the optimal covariance can be calculated. The accuracy and reliability improvements achieved by considering the noise correlations were validated through a dynamic simulation and an experiment. The optimal covariance bounding through the proposed algorithm is expected to improve the extended receiver autonomous integrity monitoring-based performance, which is related to vehicle safety [29]. Furthermore, if higher TDCP accuracy can be achieved by using the differential correction information, further improvement in the performance of the proposed algorithm and more precise navigation can be expected.

## Figures and Tables

**Table 1:** Filter configurations.

	Conventional Filter (Modified *H*)	Delayed State Filter (Modified *H*, *R*, and *C*)
Filter Configuration	$\begin{array}{l} {H^{\prime }}_{k+1}=H+J\Phi_{i+99,i}^{-1}\delta x_{i+100}\\ {R^{\prime }}_{k+1}=R\\ C_{k+1}=0 \end{array}$	$\begin{array}{l} {H^{\prime }}_{k+1}=H+J\Phi_{i+99,i}^{-1}\delta x_{i+100}\\ {R^{\prime }}_{k+1}=R+\sum_{j=0}^{99}J\Phi_{i+j,i}^{-1}Q{\left(\Phi_{i+j,i}^{-1}\right)}^{T}J^{T}\\ C_{k+1}=-\sum_{j=0}^{99}\Phi_{i+99,i}\Phi_{i+j,i}^{-1}Q{\left(\Phi_{i+j,i}^{-1}\right)}^{T}J^{T} \end{array}$

**Table 2:** Noise analysis results (engine on/off).

Noise (σ)	Accelerometer (m/s^2^)			Gyroscope (°/s)		
	x-axis	y-axis	z-axis	x-axis	y-axis	z-axis
Engine Off (Stop)	0.0118	0.0111	0.0119	0.0547	0.0502	0.0448
Engine On (Stop)	0.1095	0.1203	0.0433	0.2659	0.2633	0.0562

**Table 3:** Horizontal results (simulation).

Meter	IMU Sensor Case		2.5× Case		5× Case	
	3σ	Accuracy	3σ	Accuracy	3σ	Accuracy
Conventional Filter	0.469 m	0.168 m	0.779 m	0.214 m	1.410 m	0.274 m
Delayed State Filter	0.384 m	0.138 m	0.390 m	0.139 m	0.412 m	0.123 m

**Table 4:** Computational load comparison.

**Time (s)**	**Conventional Filter**	**Delayed State Filter**
	$8.2\times 10^{-5}$	$10.6\times 10^{-5}$

**Table 5:** Horizontal results (Monte Carlo simulation).

Meter	Conventional Filter		Delayed State Filter	
	3σ	Accuracy (RMS ^1^)	3σ	Accuracy (RMS ^1^)
Horizontal	1.411 m	0.255 m	0.417 m	0.142 m

**^1^** Root mean square (RMS).

**Table 6:** Horizontal results (experiment).

Meter	IMU Sensor Case		2.5× Case		5× Case	
	3σ	Accuracy	3σ	Accuracy	3σ	Accuracy
Conventional Filter	0.691 m	0.485 m	0.925 m	0.486 m	1.486 m	0.486 m
Delayed State Filter	0.636 m	0.449 m	0.638 m	0.442 m	0.642 m	0.439 m
